# Exploiting Temporal Features in Calculating Automated Morphological Properties of Spiky Nanoparticles Using Deep Learning

**DOI:** 10.3390/s24206541

**Published:** 2024-10-10

**Authors:** Muhammad Aasim Rafique

**Affiliations:** Department of Information Systems, College of Computer Sciences & Information Technology, King Faisal University, P.O. Box 400, Al-Ahsa 31982, Saudi Arabia; mrafique@kfu.edu.sa

**Keywords:** nanoparticle morphology, spatiotemporal network, semantic segmentation

## Abstract

Object segmentation in images is typically spatial and focuses on the spatial coherence of pixels. Nanoparticles in electron microscopy images are also segmented frame by frame, with subsequent morphological analysis. However, morphological analysis is inherently sequential, and a temporal regularity is evident in the process. In this study, we extend the spatially focused morphological analysis by incorporating a fusion of hard and soft inductive bias from sequential machine learning techniques to account for temporal relationships. Previously, spiky Au nanoparticles (Au-SNPs) in electron microscopy images were analyzed, and their morphological properties were automatically generated using a hourglass convolutional neural network architecture. In this study, recurrent layers are integrated to capture the natural, sequential growth of the particles. The network is trained with a spike-focused loss function. Continuous segmentation of the images explores the regressive relationships among natural growth features, generating morphological statistics of the nanoparticles. This study comprehensively evaluates the proposed approach by comparing the results of segmentation and morphological properties analysis, demonstrating its superiority over earlier methods.

## 1. Introduction

Antonie van Leeuwenhoek’s pioneering microscopic evaluation of cells revolutionized our understanding of microorganisms, laying the groundwork for significant scientific advancements of his time. With modern advancements, imaging microscopes such as scanning tunneling microscopes (STMs), scanning electron microscopes (SEMs), and transmission electron microscopes (TEMs) now allow us to visualize nanoscale structures with remarkable detail. As Richard Feynman insightfully remarked, “There’s plenty of room at the bottom”, highlighting the vast opportunities that nanotechnology presents across all disciplines. This burgeoning field has direct applications in numerous areas highlighted by the United Nations’ sustainable development goals, including health, clean water, energy, industry innovation, and climate action [1]. The impact of nanotechnology is particularly notable in sectors such as medicine, health sciences, agriculture, energy, and environmental protection [2]. The effectiveness of nanoparticles is contingent upon their physical properties throughout various stages of synthesis and growth, making the study of their morphological properties a crucial focus in contemporary research.

The morphological properties of nanoparticles, such as shape, size, and growth, are critical in defining their characteristics and influencing their suitability for various applications. Ref. [3] provides a comprehensive analysis of nanoparticles, categorizing them based on these properties. Similarly, ref. [4] offers an in-depth evaluation of morphological features necessary for effective assessment of physicochemical parameters, underscoring the importance of precise nanoscale measurements in the advancement of nanotechnology. Additionally, the impact of morphology on a range of copolymers is explored in relation to their application in retinal cells, as detailed in [5]. Within metal-based nanoparticles, gold nanoparticles (AuNPs) are classified according to their morphology. A subcategory, the spiky Au nanoparticles (Au-SNPs), is examined for its morphological properties during growth through TEM imaging. The study by [6] advances this field by performing an automated morphological analysis of Au-SNPs, showcasing the intricate detailing possible with modern technological tools.

An accurate analysis of the morphological properties of Au-SNPs is crucial for enhancing their application across various fields. The particles’ shapes and sizes are particularly advantageous for use in biomedicine, especially in targeted drug delivery. Additionally, Au-SNPs have demonstrated effectiveness in surface plasmon resonance (SPR) sensing and surface-enhanced Raman scattering (SERS) detection. Recently, their applications have expanded into biosensing and catalysis, illustrating their versatile utility. In [6], the morphological analysis was conducted manually, a painstaking process often performed on data collected at temporally distant intervals. Such delays can lead to significant shape changes, rendering manual analysis intractable as every recording instance would need individual evaluation. Therefore, an automated method for analyzing the morphological properties of nanoparticles is indispensable. Implementing an accurate automated approach could significantly accelerate advancements in nanotechnology, making the process more efficient and reliable.

Artificial intelligence (AI) has harnessed more power using artificial neural networks (ANNs), abundant digital data, and substantial computing power [7]. This advancement targets the automation of digital tasks typically performed by computer operators, which require minimal human intelligence [8]. One such task is the morphological calculation of nanoparticles in microscopic images. This involves identifying the particle boundaries and applying parametric metrics to calculate their morphological properties [9]. Earlier attempts at automation employed conventional AI techniques. A support vector machine (SVM) with handcrafted features was used to analyze dendritic cell maturation by Lohrer et al. [10]. It uses electron microscopic images from an AFM and SEM of dendritic cells that are processed using a conventional morphological operation. Typically, these microscopic images undergo pre-processing, including noise removal, followed by feature extraction through histograms of oriented gradients (HOGs) and classification using error-correcting output codes (ECOCs).

Deep learning techniques [11,12] are the state of the art in AI and have revolutionized the automation of labor-intensive tasks. A recent study [13] evaluates multiple state-of-the-art deep learning models for the segmentation of nanoparticles in bright-field transmission electron microscopy (BF-TEM) and environmental transmission electron microscopy (E-TEM) images. The BF-TEM images are for nanocatalyst Pt nanoparticles, whereas the (E-TEM) images are for nanoparticles, and all particles exhibit regular shapes. A prominent contemporary deep learning model, the generative adversarial network (GAN), is popular for generating spatial data. The GAN loss function is used in [14] to train a deep network used for segmentation of tumor in medical images. Furthermore, another GAN-inspired network [15] utilizes a stack of hourglass networks for the segmentation of retinal blood vessels, incorporating a generator and discriminator-focused loss function to enhance performance.

Deep neural networks (DNNs) have gained popularity in the segmentation of nanoparticles within images, significantly enhancing the efficiency of morphological analysis. For instance, ref. [16] utilized an artificial neural network (ANN) for the automated analysis of metal nanoparticles in electron microscopic images. This involved segmenting the particles to calculate their morphological properties, such as size, circularity, and diameter. Similarly, a recent study employed U-Net++, an hourglass-based convolutional neural network (CNN), to segment nanoparticles in microscopic images [17]. It uses STM images and also calculates properties like circularity, size, and diameter. A recent proposal extends the techniques of automated calculation of morphological properties to the growth kinematics analysis in videos of nanoparticles. Ref. [18] uses a variant of an hourglass neural network and proposes a new loss function to focus on the peculiar structure of Au-SNPs. However, it does not consider the temporal relation of the growth kinematics in segmentation of the particle in continuous monitoring of particles in microscopic images.

This study investigates the impact of incorporating temporal cohesion in feature analysis for the automated morphological evaluation of the kinematic growth of Au-SNPs. While [18] conducted a frame-by-frame automated analysis focusing solely on spatial cohesion, this research highlights the critical role of temporal cohesion in accurately segmenting particles over sequential TEM images. To address this, a new architecture of an ANN exploiting the hard inductive bias of the convolutional layers in an hourglass network with the soft inductive bias of recurrent layers is proposed in this study. In the proposed architecture, the spatial cohesion is explored by the convolutional layers, and the temporal relationships are exploited by the recurrent layers. Moreover, the loss function with the added term used in [18] with a Dice coefficient loss [19] is tested with the proposed network’s composition. This research has the following contributions:

- A network composition favoring spatiotemporal cohesion.
- A two-fold fusion: architecture and features.
- Testing boundary loss function with the hard and soft inductive bias.
- Use of Au-SNP TEM videos for automatic morphological analysis.
- An edge device implementation of the proposed model.

The study is structured as follows: Section 2 describes the methodology of proposed approach, Section 3 outlines the experimental setup, and Section 4 provides a detailed analysis and comparison with other methods. Finally, Section 5 and Section 6 explore future directions and summarize the findings of this study.

## 2. Methodology

ANNs represent the state of the art in artificial intelligence (AI) and computer vision (CV) tasks. Specifically, deep learning (DL) techniques are continuously evolving, resulting in performance enhancements and an expanded range of applications across various domains. However, a universal DL solution that applies to all problems does not exist, as each approach must be tailored to the specific nature of the data. AI problems are typically categorized based on data type into vision, language, sensor, and tabular problems. Computer vision encompasses all tasks involving images or sequences of images that can be readily interpreted by humans. The growth of nanoparticles, observed through transmission electron microscopy, results in a series of images, forming a video. Consequently, the automatic analysis of the morphological properties of Au-SNPs is inherently a video analytics problem.

**Figure 1:**
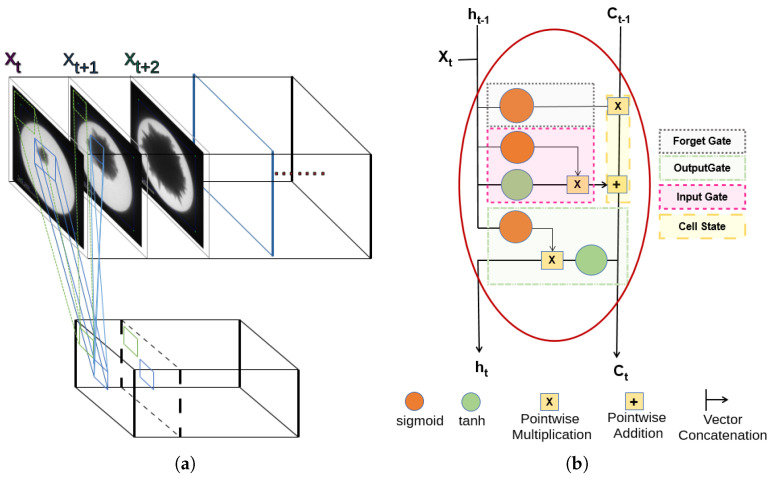
(**a**,**b**) depict the hard and soft inductive bias, where the CNN shown in (**a**) shows the spatial coherence, and the LSTM node in (**b**) depicts the temporal cohesion. (**a**) Hard inductive bias of CNN. (**b**) Temporal cohesion with an LSTM node.

CNNs [20] are deep learning networks which are the state of the art in CV for images and a first choice for video processing by breaking the video into a sequence of images and then performing feature extraction. CNNs possess a hard inductive bias which caters for the spacial coherence in the adjacent pixels in images. The inductive bias is synthesized with the connection established with the smaller grid of pixels by localizing the view of the activation function (neuron) deep in the neural network (Figure 1a depicts a CNN). This localization is cascaded layer by layer in the network and helps the network in learning the local features in the spacial data (image) while moving toward the final decision layer of the CNN. The smaller grid of pixels is often termed the window size, represented by (W), and is square in shape, which gives an impression of a subimage. Another important parameter in defining the required bias is the independence of the local spatial regions, which are usually kept overlapping and represented by a stride (S). The redundancy of the local regions in learning features in the deep layers is set with this parameter. The morphological properties of AuNPs during their growth in the TEM image are calculated by first extracting the particle from the image. Here, segmentation is a per-pixel classification problem and benefits from the inductive bias proposed in the hourglass CNN [18,21], which is a composition of an encode-decoder ANN. Figure 1a depicts a hard inductive bias with impactful parameters.

### 2.1. Soft-Inductive Bias with Recurrent Connections

The morphology of growing particles is often meaningless if calculated only in images as an exploitation of spatial features, particularly when richer information in the form of videos is available. The sequential or temporal granularities have a significant value in the correct analysis of the morphological properties. A type of ANN favoring the temporal granularity of the data is the recurrent neural network (RNN), which carries the information about features explored in the earlier time step to the next time step. A technique proposed in [22] shows the most effective hard and soft inductive biases with attention layers to explore the temporal coherence of data. However, transformers are resource-intensive, and abundant data and computer power are imperative for successful training of an ML model. Nanoparticle data are scarce, and another requirement is to use an embedded system for the inference of the morphological properties analytics. RNNs ref. [23], which are used for most AI tasks with sequential data, can be used for this purpose. Ref. [24] reinvigorates the efficacy of the RNN and discusses the attention mechanism, which is key for the transformers as a special type of RNN. It also claims the transformers are then a variant of the RNN. Figure 1b shows a long short-term memory (LSTM) [25] node in an RNN layer that effectively keeps track of temporal coherence.

**Figure 2:**
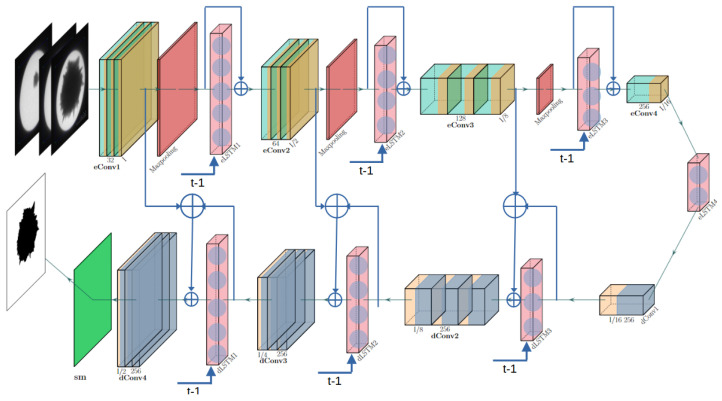
The proposed architecture of deep neural networks for segmentation. ⊕ denotes the concatenation of the features among various layers. The arrow points the direction of the features.

The growth of Au-SNPs carries cohesive features from one observation to the next observation in TEM images, and thus the inductive bias in RNN benefits the imperative analysis of morphological properties. The spatiotemporal prior in the Au-SNP growth observed with TEM images is thus a challenge considering the combination of the hard inductive bias of convolutional layers in CNN and soft inductive bias of recurrent layers in an RNN. Ref. [26] uses recurrent layers in the U-Net hierarchy by passing each down-sample layer to a recurrent layer and similarly at the up-sample layers. However, the distinction of spatial and temporal features is as important as their coherence. In this work, we propose a composition of the network which is an artistic fusion of convolutional layers interleaved with the recurrent layers, and the composition is organized in an hourglass network architecture. A novel contribution is to allow distinctive spatial features and temporal granularity fusion in the network. Figure 2 shows the composition of a complete network.

The composition of the hourglass network is the same as discussed in [18]. The difference in our proposed architecture is that convolutional layers forming the encoder and decoder in the hourglass network are interleaved with the recurrent layer. The spatial filters are passed to the LSTM (recurrent) layer, and the input to the LSTM is fused with the output of the LSTM and passed to the next convolution layer. For instance, image frame $I_{t}$ (where *t* is the frame number in the sequence) is passed as an input to the network and first passes through all modules of the encoder (*E*). Each $E_{i},i\in \left\{1,\cdots ,m\right\}$ is composed of convolutional ($E_{i}^{c}$) and recurrent ($E_{i}^{r}$) layers, and *m* is the number of modules. The following equations calculate the features from each convolutional and recurrent layer in the encoder modules, respectively:

(1) $$E_{i}^{c}=E_{i-1}^{c}⊕E_{i-1}^{r}\left(O\left(E_{i-1}^{c}\right)\right),$$

(2) $$E_{i}^{r}=O\left(E_{i-1}^{c}\right),$$

where O(·) is the output of a layer given as input, and ⊕ is a fusion of the features from different layers. In each $\left(E_{i}^{c}\right)$, the resolution of the receptive field for the spatial features is increased by twice that of each convolutional layer to the next convolutional layer, and the following [18] convolutional layers in the encoder use a kernel of size 3×3 for all convolutions. The recurrent layers have the number of LSTM nodes in decreasing order as *i* increases.

The encoder layer ends with a recurrent layer, whose output is fed to the first deconvolutional layer, which generates features contributing to the spatial resolution of the segmentation of the nanoparticle. The decoder modules are also formed by interleaving recurrent layers in deconvolutional layers. For instance, $O(E_{m}^{r})$ is passed to the decoder (*D*). Each $D_{i},i\in \left\{1,\cdots ,n\right\}$, is composed of deconvolutional ($D_{i}^{d}$) and recurrent ($D_{i}^{r}$) layers, and *n* is the number of modules. The following equations calculate the features from each deconvolutional and recurrent layer in the decoder modules, respectively:

(3) $$D_{i}^{d}=D_{i-1}^{d}⊕D_{i-1}^{r}\left(O\left(D_{i}^{d}\right)\right)⊕O\left(E_{n-i}^{c}\right),$$

(4) $$D_{i}^{r}=O\left(D_{i-1}^{d}\right),$$

where O(·) is the output of a layer given as input. The segmentation of the particle is generated by a final softmax layer classifying each pixel in a background or part of a nanoparticle, like how $softmaz(D_{n}^{d})$ classifies H×W pixels into two classes.

### 2.2. Loss Function

Nanoparticles with unique structures and shapes present significant challenges for image segmentation, even when appropriate inductive biases are applied. The loss function, which measures prediction errors, plays a crucial role in steering the learning process in the right direction. In semantic segmentation tasks, improving boundary accuracy is key to enhancing pixel-level classification. To achieve this, a Dice coefficient [27,28] is often added to the conventional cross-entropy loss. However, boundaries with sharp corners, such as those found in certain nanoparticles, require additional attention. As suggested in [18], a loss function that specifically emphasizes spikes during error calculation is more effective. The resulting loss function is a sum of cross-entropy loss, a spike-focused loss term, and the Dice coefficient presented in the following:

(5) $$L=-\sum_{1}^{2}\alpha y_{i}log\left(p\left(I_{i}^{mn}\right)\right)-1_{b}\beta log\left(p\left(I_{i}^{mn}\right)\right)+\gamma \frac{\sum_{i}p_{i}^{2}+\sum_{i}q_{i}^{2}}{2\sum_{i}p_{i}q_{i}},$$

where α,β, and γ are weights that adjust the contributions of each term in the loss function. p and q represent the probabilities of the true pixel and background pixel, respectively. This loss function was employed in this study to train the proposed spatiotemporal networks. The results indicate that incorporating the additional spike-focused loss term improves the accuracy by increasing true positives and reducing false positives, particularly in nanoparticles with complex structures like Au-SNPs.

### 2.3. Morphological Measurements

Morphological properties of nanoparticles suggest their potential use. This study focuses on two important aspects of the morphological properties of Au-SNPs. First is the aspect ratio of the shape of Au-SNPs, which can distinguish between tube-shaped, wire-shaped, and spherical nanoparticles. Second is the automatic calculation of measurements related to the particular detected shape of the nanoparticle. Moreover, particular features like spikes of Au-SNPs significantly impact the potential utilities. This study proposes an automated method that exploits the temporal coherence during the growth of the particle which, in comparison to the most recent work [18], suggests that temporal coherence generates more meaningful statistics.

The shape of the Au-SNPs is continually monitored in each available TEM image frame. The proposed DNN segment nanoparticle in the image and the particle images is processed to determine the shape of the particle. Contour detection is used in this work considering the limited scope of the shapes. The aspect ratio is calculated for the determined shape of the particle, and area and volume are calculated for the nanoparticle as required metrics. It is noted that the metrics are calculated based on the classified shape of the nanoparticle. For instance, area and volume formulas used for Au-SNP [6] are $\pi r^{2}$ and $\frac{4}{3}\pi r^{3}$, respectively, for an automatically calculated radius *r*.

## 3. Experimentation

A primary focus of this study is to improve the automated analysis of nanoparticles in a continuous stream of TEM images during their growth. The methodology devised for the analysis was evaluated with experiments performed on videos generated for the Au-SNP [6]. TEM images were captured during the process of SNP growth in an experiment performed for a few minutes, and every quarter of a second an image was taken. Once the image data were available, some images were manually segmented to create a ground truth for supervised learning of the proposed ANN. The training of the DNN was performed on a machine with an Intel Corei7 processor, 16 GB of RAM, and an Nvidia GeForce RTX 3070Ti GPU. Moreover, the experiments were designed to run the inference on an embedded system. The inference was performed on an Nvidia Jetson nano-embedded board. The performance on the embedded board was sufficient for the imaging setup, where images were captured at 4 frames per second (FPS).

### Spatiotemporal Neural Network

The proposed ANN was trained using a supervised learning algorithm with labeled data. Unlike the dataset used in [18], which comprised fewer images with labels captured at temporally distant intervals, this study utilizes a continuous stream of images where frames are labeled at closer intervals. This approach aims to capture the inherent temporal connectedness during nanoparticle growth. This continuous data stream contrasts with previous techniques that analyzed each image independently and presented additional challenges during training due to the need for sequential pattern recognition. Since the proposed approach employs layers that capture sequential patterns in the data, it requires a continuous stream of images along with corresponding ground truth labels. The DNN was trained using sequential frames extracted from video footage, with manual labeling of each frame. This study employed DNN models with both continuous frame-by-frame input and a frame-skipping method, where inputs were provided with a two-frame gap. The inference accuracy difference between these methods was negligible, leading to a decision not to present the results separately. Consequently, the results discussed in this paper are based on the model trained using the frame-skipping approach.

**Figure 3:**
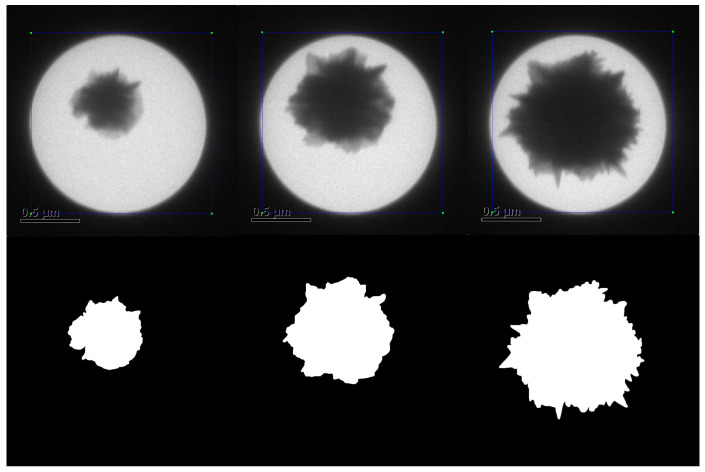
The sample of images for Au-SNP growth monitored in TEM images. The top row shows the images for frame no. 1123, 1156, and 1180 (left to right), and the bottom row depicts the ground truth.

Figure 3 shows a sequence of images used for the experiments, and it is evident that the spikes in a region have a temporal relationship between the previous image frame and the next. A lucid coherence in the growth of the nanoparticle is discernible, which impacts the calculation of correct morphology and also helps to reduce noise. The hyperparameters for the DNN given in Figure 2 are as follows: *m* and *n* are equal to 4 for the network tested in this study and can be appropriately adjusted for the problem in hand. Convolution layers in the encoder (*E*) use a square spatial filter (kernel size) of size 3×3, a stride of 1, and no padding in layers of the encoder. The decoder uses deconvolution layers with the kernel size to regenerate images with the same size as those of the input. Each recurrent layer in the encoder of the network starts with 1024 neurons, which is reduced to half in subsequent layers, and vice versa for the decoder. The dataset is split into testing and training using a defined split to feed the recurrent layers, which includes one frame from each second of the collected images. Also, for a fair comparison, the images reported by [18] in the results are not included in the training split. The RAdam [29] optimizer was used for parameter adjustment. The optimizer uses a learning rate $lr=1\times 10^{-3}$, $\beta_{1}=0.9$, $\beta_{2}=0.999$, a weight decay λ=0, and $\epsilon =1\times 10^{-8}$.

Morphological properties were calculated after analyzing the segmented nanoparticles from the images. Three shapes were targeted in this study, but the data contain only one available shape. Testing with cylindrical and ellipsoidal shapes was left for future study with available synthetic data. The numbers of spikes were also estimated following the procedure discussed in [18].

## 4. Results

This study comprehensively evaluated the proposed method with various metrics. First, this study automatically estimated the morphological properties of Au-SNPs. The morphological properties include the shape of the particles, which was estimated from the analytics performed on the segmented nanoparticle using the DNN. The shape was estimated at each available frame, which is effective in applications where the deformation of the particle is not desired. The area and volume of the particles were also calculated from the segmented particle as the Au-SNP is spherical in shape with spikes on the surface. The area and volume are essential in reflecting the uniform growth of the particle. The spikes are an essential component of a Au-SNP, so the number of spikes was also recorded in this study. Second, the segmentation quality of the proposed DNN was evaluated using the commonly used metrics of IoU and F1 scores. Moreover, the DNN was tested with qualitative comparisons.

**Figure 4:**
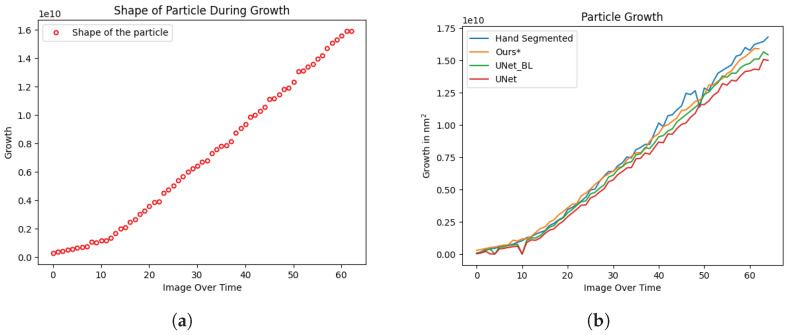
(**a**,**b**) depict the identified shape and the area of a Au-SNP during its growth. (**a**) Shape. (**b**) Number of spikes.

**Figure 5:**
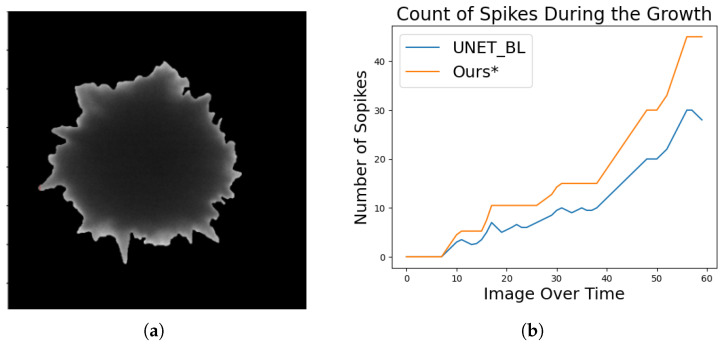
(**a**) shows the masked particle after segmenting the image, (**b**) shows the spike count graph. (**a**) Masked particle. (**b**) Spike count.

The morphology of a Au-SNP during its course of growth captured in TEM images is automatically estimated by identifying its shape, and Figure 4a depicts the shape. The red circles represent a circular shape of the particle in a region, and the aspect ratio of the identified region is close to 1:1 during its growth. Figure 4b shows the area of the particle during its growth. It is clearly visible that some miscalculations, which are present in the results produced by earlier techniques, were not performed by the DNN proposed in this study. The temporal coherence takes into account the results from the previous time steps, and its impact helps to alleviate the errors caused by predicting the sequential data with hard inductive bias in a spatial ANN alone. Another interesting observation is the uniformity in estimating the number of spikes as compared to the earlier presented techniques. Figure 5b depicts a chart comparing the difference in the results produced by earlier techniques in [18], and the impact is a direct effect of the DNN proposed in this study as the count estimation method is the same for both. This study used an additional prepossessing step by employing a threshold to calculate spikes at two stages by mapping masked segmented nanoparticles to the original particle as shown in Figure 5a. It is evident that this improves the results.

**Figure 6:**
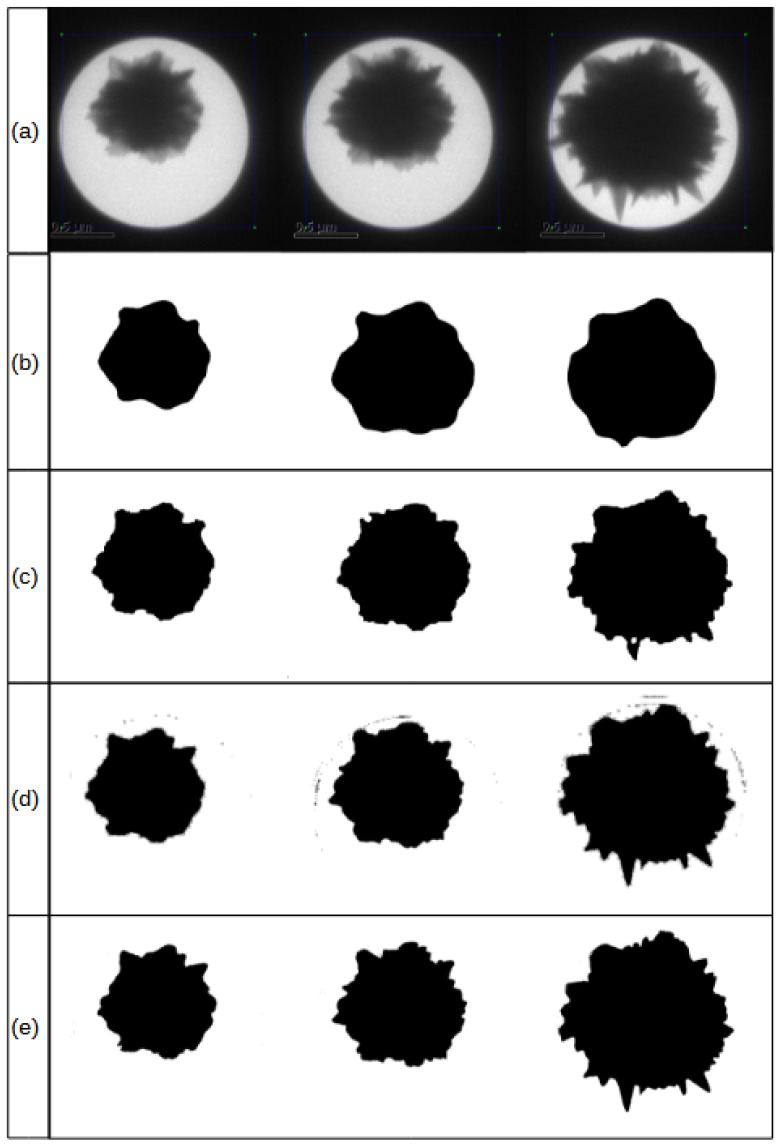
The qualitative results of segmentation from various techniques. Row (**a**) is the original image of the particle, row (**b**) shows segmentation results of the MaskRCNN, row (**c**) shows segmentation results of conventional techniques used in [9], row (**d**) shows the results of [18], and row (**e**) shows results generated by our proposed technique.

Figure 6 depicts the qualitative results of nanoparticle segmentation using three existing techniques: Mask R-CNN [30], conventional methods [9], and a spatial-only hourglass network [18]. Each method demonstrates challenges in preserving the temporal correlation essential for precise nanoparticle segmentation. the MaskRCNN, due to its inductive bias towards color-based coherence, struggles to accurately capture the spikes that are crucial for defining the nanoparticle’s characteristics and determining its potential applications.

While conventional methods, enhanced with explicit domain knowledge, show improved performance, they still encounter significant challenges. Notably, they occasionally generate voids within the nanoparticle, which post-processing cannot reliably rectify, as morphological operations risk altering the spikes. The spatial-only hourglass network [18] shows improvements over prior techniques but falls short in maintaining temporal coherence during particle growth, as it operates on a frame-by-frame basis. This limitation is apparent in Figure 4, where spike growth lacks stability, and in Figure 5, where the number of spikes appears inconsistent.

It is evident from the results that exploiting the temporal coherence in the sequence of frames captured during the growth of a nanoparticle improves the segmentation results. The impact is twofold: First, it improves the true positives of the growing regions in the particle, particularly around the spikes, and second it reduces the noise in the segmentation results which is visible in earlier frame-by-frame segmentation results presented in [18]. These effects are a direct impact of the recurrent layers used in the network in addition to the convolution and deconvolution layers of hourglass networks. The impact of the fusion of features from the convolution and the recurrent layers is another factor that improves the results. Furthermore, the segmentation is analyzed using quantitative evaluation, and Table 1 shows the IoU, F-Score, precision, and recall of the proposed methods. It is clearly evident from the figures that the ideas used in this study improved the true positives and reduced the false positives. There is a slight impact on the efficiency, which is slightly reduced.

Although the MaskRCNN has high FPS, it lacks the correct segmentation of spikes. The proposed approach is competitive in speed and is more practical given the specification of imaging hardware, such as the used electron microscope, which captures images at a maximum of 4 FPS. Furthermore, by incorporating sequence-aware layers, our method allows us to skip frames while still retaining crucial temporal information, unlike image-only techniques that lack this capability.

## 5. Discussion

This study focuses on Au-SNPs because their spatially complex structure presents significant challenges for morphological analysis with existing techniques. Besides other innovations, this research addresses two primary limitations of previous methods: first, the integration of temporal coherence during nanoparticle growth, and second, the precise modeling of spike growth as part of the morphological analysis. While most nanoparticles possess simpler shapes and are often effectively analyzed using conventional DNN segmentation techniques, typically frame-by-frame segmentation, there is a notable gap in growth analysis using video data due to limited data availability. The proposed approach is designed to accommodate a broader array of nanoparticle shapes, supporting both temporal and spatial data modalities. By leveraging a combination of recurrent and convolutional layers, the network is adept at processing and analyzing both temporal and spatial data, providing a comprehensive picture of nanoparticle growth dynamics.

Moreover, the ablation study is embedded in the comparison of methods, as previous techniques relied solely on CNN layers. Additionally, the experiments tested an approach using only an RNN for this study. However, the results were not substantially different and thus are not included here. Directly employing recurrent layers with image data poses challenges due to the high number of individual pixel values, complicating their practical application.

It is essential to recognize that the schematics of Au-SNPs, as referenced in [6], present a limitation when viewed in 2D electron microscopic images. These images only capture a partial view of the 3D structure, offering an approximation of the visible side of the particle’s morphology. The thresholding method to count the number of spikes at two levels is a workaround. Future advancements in electron microscopy are expected to enhance our ability to visualize particles in complete 3D detail. In parallel, an extension of the current work is the use of 2D images to generate 3D Au-SNPs. As claimed in [24], RNNs are a general form of transformers that are the state of the art in generative machine learning. The 3D structure of a Au-SNP is provided in [6]. However, the current data availability is insufficient for effectively training a DNN. The recent literature has introduced generative models aimed at reconstructing 3D objects from point cloud data, partial structures, and 2D images. Despite these advancements, such models still demand a substantial volume of 3D structural data to effectively train a DNN [31,32].

For future work, this study could be extended by generating synthetic data based on the nanoparticle shapes discussed in [6]. These shapes could be transformed into suitable 3D object formats, creating a comprehensive dataset to train the network. The DNN could be trained across several modalities, such as transforming 2D images into 3D shapes, converting partial views to complete 3D structures, or generating 3D shapes from a dual-particle input. Another promising direction is to simulate the growth of nanoparticles over time from an initial particle, subsequently generating electron microscopic images to identify any anomalous growth patterns. This approach offers valuable insights for 3D modeling of nanoparticles. Additionally, developing an end-to-end network, as suggested in [33], is contemplated as future research, especially as more particle samples become available.

## 6. Conclusions

The growth of nanoparticles is a temporally cohesive process, and TEM video data that capture this growth should be analyzed using AI techniques with inductive biases that support sequential events. This study explores this hypothesis by introducing a novel architecture that combines hard and soft inductive biases in an artificial neural network (ANN). The proposed deep neural network (DNN) technique was applied to the automatic morphological analysis of Au-SNPs, which was previously performed using a spatially focused CNN. The results demonstrate that a combination of convolutional and recurrent layers, along with an optimal fusion of features, effectively captures the temporal and spatial relationships in the data. Additionally, this study highlights the positive impact of the use of segmented particles with the proposed DNN for the automatic analysis of morphological properties of nanoparticles. The approach can be further extended to generate 3D shapes of nanoparticles, potentially reducing the need for complex and expensive experiments to grow nanoparticles for property analysis.

## Figures and Tables

**Table 1:** Quantitative results of the segmentation. (↑ indicates the higher the better).

Technique	Time (FPS) ↑	IOU ↑	F1-Score ↑	Precision ↑	Recall ↑
MaskRCNN [30]	**10**	0.971	0.982	0.973	0.988
U-Net	3	0.986	0.992	0.991	0.994
U-Net-BL [18]	3	0.987	0.992	0.994	0.991
Ours	2.5	**0.993**	**0.995**	**0.994**	**0.996**

## Data Availability

The original contributions presented in the study are included in the article, further inquiries can be directed to the corresponding author.

## Abbreviations

The following abbreviations are used in this manuscript:

ANN	Artificial neural network
DNN	Deep neural network
DL	Deep learning
SNP	Spiky nanoparticle
CNN	Convolutional neural network
RNN	Recurrent neural network
